# Diagnosis and Management of Scalp Metastases: A Review

**DOI:** 10.3390/diagnostics14151638

**Published:** 2024-07-30

**Authors:** Stephano Cedirian, Luca Rapparini, Andrea Sechi, Bianca Maria Piraccini, Michela Starace

**Affiliations:** 1 Dermatology Unit, IRCCS Azienda Ospedaliero-Universitaria di Bologna, 40138 Bologna, Italy; stephano.cedirian@studio.unibo.it (S.C.); biancamaria.piraccini@unibo.it (B.M.P.); michela.starace2@unibo.it (M.S.); 2 Department of Medical and Surgical Sciences, Alma Mater Studiorum University of Bologna, 40138 Bologna, Italy; 3 Dermatology Unit, Fondazione IRCCS Ca’ Granda Ospedale Maggiore Policlinico, 20122 Milan, Italy; sechi.andre@gmail.com

**Keywords:** scalp metastases, cancer, dermoscopy, trichoscopy, imaging, oncodermatology, histopathology, management, prognosis

## Abstract

Scalp metastases (SMs) are particularly noteworthy, representing around 4–7% of cutaneous neoplasms in this region of the body, possibly due to its rich blood supply. Diagnosis of SMs involves a systematic approach encompassing oncologic history, clinical examination, dermoscopy, imaging, and histopathological assessment. Clinical presentations of SM can vary, but dermoscopy reveals unique vascular patterns aiding in diagnosis. Imaging, particularly MRI and CT, and histopathological evaluation are mandatory for definitive diagnosis. Treatment strategies vary depending on tumor characteristics and staging, ranging from surgical excision to systemic therapies like chemotherapy or radiotherapy. Multimodal approaches tailored to individual cases yield optimal outcomes. The diagnostic tools available do not always allow SMs to be diagnosed, and often the lack of knowledge on the part of oncologists in suspecting SMs can delay an early diagnosis. This review provides clinicians with a practical guide for the timely diagnosis and management of SM, emphasizing the importance of a multidisciplinary approach and personalized treatment strategies for improved patient outcomes.

## 1. Introduction

Cutaneous metastases (CMs) are not a common phenomenon: it has been reported that they occur in an estimated range of 0.5% to 10.4% of all cancer patients [1,2,3,4,5,6,7,8]. Various mechanisms facilitate the metastasis of cancers to the skin, including direct invasion, implantation along a surgical scar, and spreading through the lymphatic or hematogenous routes [9,10]. CMs are indicative of a poor prognosis and can manifest as either the first clinical sign of the disease or as a sign of recurrence following unsuccessful treatment [11,12].

The scalp is a distinctive anatomical region that accounts for nearly 2% of cutaneous neoplasms [13], with CMs comprising around 4–7% of these cases [14]. This increased prevalence of CMs on the scalp may be attributed to its abundant blood supply compared to other body areas [15]. Common primary tumors associated with scalp metastases (SMs) originate mostly from the gastrointestinal (GI) tract, lungs, prostate, and breast [14,15,16,17,18]. Paolino et al. conducted a systematic review, describing the main demographic features of SMs [17]. In 118 reports, accounting for 123 patients, they found out that women were disproportionately affected by SMs compared to men, accounting for 53.7% of cases compared to 46.3% in men, possibly due to the disproportion of breast cancer among women and men. The GI tract was the most common site of the primary tumor, representing 24.4% of cases, followed by the breast (17.9%), kidney (8.1%), lung (7.3%), thyroid (7.3%), uterus (6.5%), central nervous system (6.5%), liver (3.3%), and other anatomical areas, which accounted for 18.7% of cases [17].

In a recently multi-center retrospective study, the general and dermoscopic characteristics of CM in 583 patients were described, showing that the head and neck area was a common site, and that the most frequently involved primary tumors were melanoma and breast cancer [8].

Literature concerning SMs remains limited, primarily comprising case reports, case series, and retrospective studies [Table 1] [14,15,16,17,19,20,21,22,23,24,25,26,27,28,29,30,31,32,33]. In addition, confusion could be reported using interchangeably the term “Neoplastic Alopecia” to refer to SMs [16,17], although this study highlights a nuanced distinction. Indeed, SMs may manifest with various lesion types, not always involving alopecic patches, underscoring the necessity for differentiation [19,20,21,22]. On the contrary, neoplastic alopecia can be manifest as a diffuse alopecia without alopecic patches [15].

We conducted a narrative review of the literature to explore the diagnostic approaches and management of SMs, offering clinicians a practical guide to deal with this condition.

## 2. Diagnosis

The diagnostic strategy for SMs encompasses a systematic, multi-step process, requiring a comprehensive understanding of the patient’s oncologic history, precise clinical identification of SM, thorough dermoscopic assessment, imaging techniques, and histopathological examination.

SMs typically occur after the diagnosis of the primary tumor in most cases, while a small percentage occur before the primary malignancy is diagnosed and it represents the diagnostic site of the tumor [1,2,17]. In a significant proportion of cases, the diagnosis of SMs coincides with the diagnosis of the primary tumor, particularly in GI cancer cases; this may be linked with the delayed diagnosis of GI tract tumors, often presenting with minimal or absent symptoms in the early stages, thus making distant metastatic events the initial indicators of malignancy that can be very aggressive [17].

Diagnosing SM can be difficult, particularly when it occurs before the diagnosis of primary malignancy. The differential diagnosis encompasses several conditions such as pilar cyst, epidermoid cyst, keratoacanthoma, cutaneous squamous cell carcinoma, basal cell carcinoma, atypical fibroxantoma, primary cutaneous melanoma, Merkel cell carcinoma, chronic radiation dermatitis, discoid lupus erythematosus, central centrifugal scarring alopecia, scalp sarcoidosis, lichen planopilaris, pseudopelade of Brocq, and alopecia areata among other manifestations [2,16,34,35].

While clinical, dermoscopic, and imaging steps aid in recognizing suspicious lesions, histopathology remains mandatory whenever an atypical scalp lesion is encountered [2,15,36,37].

### 2.1. Clinical Features

Clinical presentation is protean and encompass several factors, including elementary lesion, size and color, localization within the scalp, and associated cutaneous finding within or outside the scalp.

Elementary lesion: SMs exhibit a broad array of morphological presentations, ranging from papules/plaques, nodules, and ulcers to inflammatory eruptions [1,2]. Typically, they may be asymptomatic, itchy or painful [1,2,16,38]. SMs present mostly as single lesions although sometimes there might be multiple lesions [16,39,40,41,42,43,44].

Size and color: Paolino et al. delineated 11 clinical subtypes of SMs, integrating criteria such as clinical appearance, color, and maximum diameter [17]. Red/violaceous nodules were the most commonly observed subtype, with an average diameter of 2.2 ± 1.2 cm, followed by flesh-colored nodules with an average diameter of 3.4 ± 4.5 cm. The red appearance of these lesions may be attributed to the rich blood supply of the scalp [17,45,46]. Interestingly, the remaining nine subtypes were less frequently reported, with larger average diameters compared to nodular red/violaceous and flesh-colored lesions. Single lesions are predominant in the red/violaceous and uncommon subtypes, but not in flesh-colored nodular lesions [17].

Localization within the scalp: regarding scalp site distribution, they observed no specific preference for flesh-colored nodules, while red/violaceous nodules were more commonly found in the parietal area, either alone or in combination with other sites. Uncommon subtypes were more prevalent in the frontal area, followed by parietal and occipital areas with nine and seven cases, respectively [17]. Embryonic distinction could potentially account for the observed patterning differences in SM. The dermis of the frontal and parietal scalp originates from neural crest cells, while that of the occipital and temporal scalp is derived from mesoderm. Regulatory genes, growth factors, and cellular interactions specific to these embryonic origins may influence the biological characteristics in each scalp region, causing different patterning of SMs [17].

Associated cutaneous finding within or outside the scalp: in rare instances, SM has been observed alongside distinctive skin lesions linked to the primary neoplasm [21,47]: for example, a case by Dobson et al. showed a patient that displayed telangiectatic lesions on the forehead, nose, and cheeks, while in another case report by Brasanac et al., a patient exhibited a zosteriform eruption on the trunk [48,49]. These manifestations occurred concurrently with areas of alopecia caused by SM, a condition also termed secondary neoplastic alopecia (SNA) [16]. The pathogenetic mechanism of SNA remains incompletely understood. It typically presents as a scarring process, leading to the prevailing theory that tumor cells induce a desmoplastic reaction, ultimately resulting in the destruction of pilosebaceous units. Certain fibrogenic cytokines, including basic fibroblast growth factor, transforming growth factor beta, and interleukins 4 and 6, released by neoplastic cells are proposed by some authors to contribute to follicular atrophy, resembling mechanisms observed in inflammatory alopecia [16,45,50,51,52].

### 2.2. Dermoscopy

The literature on dermoscopic assessment of SM remains limited, primarily comprising case reports and retrospective studies. The dermoscopic assessment should always provide the following information:

- Dermoscopy of vascular and/or pigmented features of the lesion;
- Dermoscopy of the related scalp changes:
  (a) Preservation versus disruption of the hair follicles and associated changes (follicular patterns);
  (b) Interfollicular changes, including color changes, scaling, and vessels (interfollicular patterns);
  (c) Changes of the growing hair shaft.

**Figure 1:**
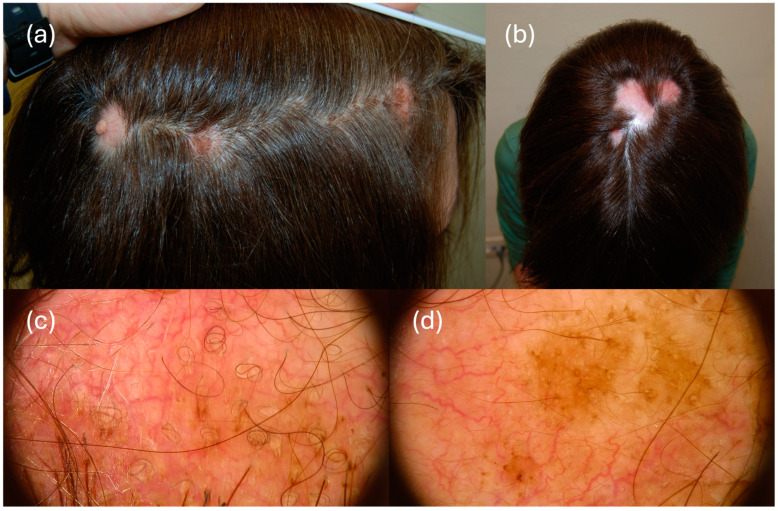
Clinical (**a**,**b**) and dermoscopic (**c**,**d**) image of a SM in a 49-year-old female patient affected by breast cancer. SM presented as multiple, infiltrated, rounded, and edematous alopecic patches, with sharp confluent margins, red in color. Dermoscopy (20×) shows diffuse erythema with a polymorphous vascular pattern, with irregularly shaped dilated, serpentine, and polymorphic vessels. Circle hairs and diffuse vellus hairs in some patches are present.

Vezzoni et al. identified distinctive dermoscopic characteristics in an 85-year-old woman with breast cancer SM, including prominent large branching blood vessels alongside slight red capillaries on a pinkish-white base. They also noted a unique orangish lesion with varied vessel patterns, surrounded by a yellow-white crust [16]. Similarly, AlSubait observed different vessel formations, such as dotted, linear, and serpentine patterns, along with a featureless white area on a pink-to-red background in a 79-year-old man with sigmoid adenocarcinoma SM [53]. These findings parallel those reported by Cetinarslan et al. in a 49-year-old man with sarcomatoid renal cell carcinoma SMs, which included white scales, varied vessel shapes, and a yellowish, structureless region on a light red background [54]. Lastly, Ravaioli et al. underscored how SM could be a potential cause of black dots at scalp dermoscopy examination [55]. The presence of atypical vascular patterns (e.g., serpentine vessels), accompanied by scaling and a white-pink background, appears to be a common feature of SMs of non-pigmented primary tumors [Figure 1]. However, it is crucial to conduct further research to establish more specific criteria in this domain.

Stanganelli et al. noted specific dermoscopic criteria in melanoma SMs, such as aggregated globules (both regular and irregular in shape), irregular streaks, structureless blue or brown pigmentation, blue-white veil, areas of regression, areas of hypopigmentation (both perifollicular and multifocal), blotches, and atypical vessels [36]. In general, melanoma metastases may reveal blue nevus-like, angioma-like, vascular, and unspecific dermoscopic patterns [56].

Dermoscopic evaluation of the vessels may also help in the differential diagnosis with other common primary skin tumors, such cutaneous squamous cell carcinoma, which showed ‘coils’ and ‘looped-hairpin vessels’ [57], and basal cell carcinoma, with ‘arborizing vessels’ represented by a large diameter, branching irregularly into finest terminal capillaries [57,58]. Moscarella et al. described the dermoscopy of atypical fibroxanthoma, characterized by white and red areas without structure and irregular linear vessels, but not serpentine vessels typical of SMs [59].

### 2.3. Imaging

Imaging plays a pivotal role in several aspects of managing scalp malignancies [37,60]. Firstly, it aids in assessing the extent of tumor invasion within the scalp [60]. Secondly, it facilitates disease staging, providing crucial information for treatment planning. Additionally, imaging guides surgical biopsy and/or resection of the tumor, aiding in precise localization and minimizing collateral damage [60]. Furthermore, it is instrumental in preoperative planning and post-treatment surveillance of scalp tumors, enabling clinicians to monitor response to therapy and detect any signs of recurrence early on [60]. Given the complex and aggressive nature of scalp malignancies, an interdisciplinary treatment approach is indispensable for their effective management [37,60].

The histological layers of the scalp can be differentiated on MRI and CT into three main categories: the skin (epidermis/dermis), subcutaneous tissue, and galea/subgalea/periosteum [37,61]. Having an understanding of the radiographic depiction of the histological layers of the scalp is crucial for accurately assessing the precise location and extent of tumors in this anatomical region [37,61].

Due to its remarkable soft tissue contrast, MRI stands as the gold standard imaging modality for assessing scalp tumors regarding their internal structure and the extent of invasion into perineural tissues and bone marrow [62,63]. Additionally, modern MRI techniques like diffusion-weighted imaging (DWI) provides valuable insights into tumor characteristics by mapping molecular diffusion, particularly water movement [60]. Malignant scalp tumors typically exhibit lower apparent diffusion coefficient (ADC) values and faster contrast material uptake compared to benign tumors, reflecting differences in cellularity and proliferation. The lower ADC value is due to the restricted movement of water molecules in neoplastic tissue caused by higher cellular proliferation [62]. For instance, Wu et al. documented a unique case of SM associated with anaplastic oligodendroglioma, which emerged six years following the surgical removal of the primary cancer [64]. MRI examination unveiled a uniformly enhanced nodular lesion with restricted diffusion in the subcutaneous tissue of the right frontal scalp [64]. Histological analysis confirmed the lesion as SM originating from the primary cancer, largely attributed to tumor seeding during the initial surgery [64,65].

Conversely, CT is more effective for visualizing bone structures and assessing direct infiltration of malignant tumors into bone tissue [63]. Subasinghe et al. documented a case involving a 56-year-old male with SM originating from hepatocellular carcinoma. The patient presented with a painless occipital scalp lump that had been progressively enlarging over three months. Contrast CT scan revealed a scalp mass accompanied by destruction of the nearby skull vault and extension into the intracranial region, although there was no penetration of the meninges [66].

Thus, the choice between MRI and CT depends on several factors, including cost, patient-specific conditions like iodine allergies, pregnancy, or renal insufficiency, and the specific diagnostic requirements of the clinician [37,63].

Additional imaging modalities such as ultrasound and positron emission tomography/CT (PET/CT) are also available [63]. High-frequency ultrasonography offers the advantage of a submillimeter resolution, with an axial spatial resolution of 100 µm at 15–18 MHz and 30 µm at 70 MHz; it serves as the primary imaging method for assessing superficial head and neck masses, particularly due to its safety profile in avoiding ionizing radiation and iodinated contrast material, making it preferable for children and pregnant women [67]. However, its main limitation lies in its limited tissue penetration, primarily allowing assessment of cutaneous and subcutaneous lesions [60]. More specifically, it offers a rapid and cost-effective means of evaluating scalp lesions, including their size, internal characteristics, vascularity, and surrounding anatomy [67]. Additionally, ultrasound can aid in directing subsequent imaging and treatment strategies, such as tissue sampling or surgical intervention [67]. Sonography of scalp cutaneous malignancies typically reveals hypoechoic solid lesions with a notable increase in vascularity [68].

Fluorine 18 fluorodeoxyglucose (FDG) PET is frequently employed for tumor grading and staging in the head and neck region [69]. Additionally, FDG-PET plays a vital role in monitoring disease progression post-treatment, particularly for identifying tumor recurrence and distant metastasis [69]. Nevertheless, the presence of infections and inflammatory conditions can lead to false-positive FDG uptake, thereby reducing its diagnostic accuracy [69]. SM frequently present as areas of increased FDG uptake, appearing as thickened skin or subcutaneous nodules on PET/CT scans [4,70].

Following the diagnosis of SM, patients typically undergo restaging through CT scans or, if accessible, PET/CT scans. This comprehensive approach aims to detect any additional visceral metastases or assess for potential diagnosis (or recurrence) of the primary disease [20,37,71,72,73].

### 2.4. Cytology

Fine-needle aspiration cytology (FNAC) is a quick, cost-efficient, and minimally invasive method particularly suitable for diagnosing superficial scalp lesions [74,75]. It facilitates efficient handling of patient samples, enabling ancillary testing such as microbiology cultures, flow cytometry for potential lymphoma cases, or obtaining additional passes for cell block preparation to conduct immunohistochemical and molecular studies [76]. In a retrospective study by Sharma et al. involving 896 cases of scalp lesions that underwent FNAC, 11 cases were identified as SMs [77]. Among these, for example, an aspirate from a scalp swelling in a known case of follicular carcinoma thyroid exhibited a tumoral lesion with cells arranged in a characteristic micro-follicular pattern, suggestive of a metastatic tumor originating from the thyroid [77].

FNAC proves to be beneficial for the differential diagnosis of SM. Malignant adnexal tumors often manifest with prolonged duration and superficial ulceration [76]. Notably, cells with coarse vacuolated cytoplasm and starry nuclei (mulberry cells) suggest sebaceous differentiation [78]. Follicular differentiation should be considered if the tumor is connected to normal follicular structures and exhibits peripheral palisading with adjacent papillary mesenchymal bodies [78]. Tumors displaying apocrine differentiation typically demonstrate granular eosinophilic cytoplasm and a single prominent nucleolus [76].

### 2.5. Histopathology

In terms of histopathological appearance, CMs typically spare the epidermis and often present with a “bottom heavy” morphology, forming pyramidal or broad-based structures [6]. Different morphological patterns such as nodular, infiltrative, diffuse, and intravascular have been noted, all showing a dermal predominance [79]. Identifying CM can be challenging in cases with predominantly superficial and epidermal architecture [1].

**Figure 2:**
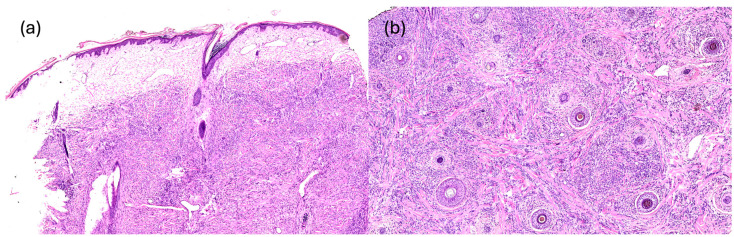
Full-thickness dermal infiltration by carcinoma of solid-cord structure and intermediate nuclear grade. SM of breast origin. (**a**) Vertical section (Haematoxylin and Eosin, 5×). (**b**) Horizontal section (Haematoxylin and Eosin, 3×).

SMs originate from different kinds of primitive tumor and histology may vary according to the primary cancer. Abdulraheem et al. reported a scenario where a 47-year-old woman previously diagnosed with invasive ductal carcinoma (showing positive results for estrogen receptor [95%] and progesterone receptor [5%]) developed scalp nodules revealing metastatic invasive ductal carcinoma with specific biomarkers. These included positive readings for ER (95%), PR (5%), cytokeratin-7 (CK-7), and GATA binding protein 3 (GATA-3), while P63 and KIT (CD117) were absent in the tumor cells and HER-2/neu score was zero [80]. When dealing with metastatic breast cancer, especially ductal carcinoma, distinct features such as a discrete nodular infiltrate of neoplastic cells extending from the superficial dermis to the underlying subcutis with intralesional fibrosis might be observed under low magnification [1]. On the other hand, lobular carcinoma often maintains a characteristic single-cell, single-file, linear, and infiltrating pattern [1]. To differentiate metastatic breast cancer from primary cutaneous adnexal carcinoma, an initial immunohistochemical panel may include markers like p63, CK5, CK14, CK17, and mammaglobin. However, it is important to note that GATA3 and CK7 can be positive in both breast and adnexal neoplasms, posing a potential challenge. Additionally, estrogen receptor and mammaglobin might not always be detected in metastatic breast carcinomas [Figure 2] [1].

Concerning SMs from lung cancer, histologically, metastases originating from lung adenocarcinoma typically exhibit moderate differentiation, appearing as solid nests, sheets, and cords of cells without connection to the epidermis [1]. They often retain certain features such as mucin deposition and sometimes show focal gland formation. Identification of the lung origin is often confirmed through immunoreactivity testing for thyroid transcription factor 1 (TTF-1) and CK7 due to the retention of a glandular component [1,81]. While Napsin A staining is common in well-differentiated lung adenocarcinoma, it lacks specificity and can also stain other tumors, such as large-cell neuroendocrine carcinomas and thyroid tumors [1,81]. Additionally, Ber-EP4 staining can lead to confusion as lung adenocarcinoma may stain positive and be mistaken for basal cell carcinoma with an insular growth pattern [82,83]. For example, Zhang et al. documented a case involving a 55-year-old male smoker with SM of poorly differentiated adenocarcinoma. Immunohistochemistry analysis revealed positive staining for CK(pan), Ki-67, CK7, CD56, and P40, while NapsinA staining was negative [35].

Primary cutaneous adnexal malignancies are the main differential diagnoses for SM [1]. In particular, malignant adnexal tumors usually exhibit focal well-differentiated, benign-appearing precursor areas [1,84]. Immunohistochemical staining panels including markers like cytokeratins (CKs) such as CK7 and CK20, SRY-related HMG-box 10 (SOX10), and p63 can aid in distinguishing among epithelioid entities. Positive staining for CK7, CK15, D2-40, and p63, and negative staining for CK20 and SOX10, typically indicate a primary cutaneous neoplasm [1,85]. On the other hand, metastatic or primary squamous cell carcinomas typically show negative staining for CK7, CK20, and SOX10 (positive in melanoma), and positive staining for p63 and CK5/6, although clinical correlation is necessary to distinguish between metastases and primary squamous carcinomas [1].

Diagnosing SM can indeed pose challenges, and being aware of its most common causes, including breast, lung, GI tract, and kidney cancers, can aid in accurate diagnosis [17]. Utilizing the appropriate immunohistochemical markers can be instrumental in differentiating between these various primary malignancies [1].

## 3. Management

Treatment for SM lacks a standardized protocol and is tailored according to the neoplasm’s receptor expression, overall staging, and patient’s comorbidities [86]. The primary treatment strategies encompass radiotherapy, chemotherapy, hormonal therapy, or other tumor-specific approaches, with the goal of potentially addressing both the tumor and its metastases [16].

In a systematic review conducted by Paolino et al. on SM, the treatment of the main tumor was documented in 84.6% of instances, whereas therapy for SM was only noted in 65.9% of cases [17]. Regarding the primary tumors, the most frequently cited treatment approach was surgical removal either as a standalone procedure or in conjunction with other modalities such as radiotherapy, chemotherapy, or both [17]. However, in the case of SM, chemotherapy emerged as the predominant treatment option, administered alone in 23 cases or combined with surgery or radiotherapy in 11 and 10 cases, respectively. Surgery followed as the next most common intervention, either alone in 24 cases or combined with chemotherapy or radiotherapy in 11 and 3 cases, respectively [17]. Given the lack of a standardized protocol for the management of SM, adopting a Multi-Disciplinary Team (MDT) approach can be helpful in defining the best therapeutic strategy for each individual patient. In addition, after the diagnosis of SM, it may be useful to perform a new instrumental staging, and in some cases a repeat liquid biopsy may also be indicated to analyze whether the tumor has mutated or developed drug resistance [87,88].

## 4. Prognosis

The presence of SM signals advanced disease and is consequently linked to a poor prognosis [11,12]. Consistently, women tend to demonstrate a higher median progression-free survival (PFS) compared to men, aligning with the overall poorer prognosis typically observed in male oncology patients [17]. Moreover, Kaplan–Meier survival analysis reveals a median overall survival (OS) of 12 months from primary tumor diagnosis and 4 months from SM diagnosis [17].

In general, tumors in the scalp are associated with a worse prognosis because this anatomical site is frequently overlooked during dermatological examinations (mainly due to hair coverage), which delays diagnosis [36,46]. Additionally, the unique anatomy of the scalp, which includes multiple layers such as the cutis with adnexa, fibroadipose tissue, lymphatic and vascular systems, and the galea aponeurotica, could contribute to the poorer prognosis of tumors compared to those in other locations [89,90,91]. Indeed, the subaponeurotic space, which separates the skull from the scalp, not only allows mobility of the scalp but may also facilitate the distant spread of tumors [92,93]. Further research is warranted to provide a deeper understanding of this aspect, as it remains controversial [36].

## 5. Conclusions

In conclusion, SM represent a challenging clinical entity often indicative of advanced disease and poor prognosis. Our review underscores the importance of a comprehensive diagnostic approach integrating clinical evaluation, dermoscopy, imaging modalities, and histopathological analysis for accurate diagnosis and staging. Understanding the diverse clinical presentations, histological features, and immunohistochemical profiles of SMs originating from different primary tumors is essential for guiding appropriate management strategies.

While treatment protocols lack standardization, multimodal approaches tailored to individual patients’ tumor characteristics, staging, and comorbidities yield the most favorable outcomes. Surgical excision, chemotherapy, radiotherapy, and targeted therapies play key roles in managing SMs, often in combination to address both primary tumors and metastases.

Continued research and collaboration are essential to further refine diagnostic and therapeutic strategies, ultimately enhancing outcomes for patients with SMs.

**Table 1:** Summary of diagnostic features and management options of cases and studies concerning SM present in literature.

First Author, Year	Study Design	Primary Tumor	Clinical Presentation	Dermoscopy	Imaging	Histopathology	Treatment
Abdulraheem et al. (2023) [80]	Case report	Breast adenocarcinoma	Erythematous, firm, non-tender, and immobile nodules	NA	MR: soft tissue nodules	Metastatic invasive ductal carcinoma. IHC: ER+, PR+, HER2−, CK7+, p63−, KIT−	Incisional biopsy. Septic shock and death
Aguiar et al. (2016) [18]	Case report	Oesophageal squamous cell carcinoma	Firm, nodular, erythematous lesions with a keratinized center, mimicking keratoacanthoma	NA	NA	Nodular dermal-based proliferation of atypical squamous cells and carcinomatous vascular emboli at the peritumoral dermis	Surgical excision. Palliative treatment
Alharbi et al. (2023) [71]	Case report	Phylloid breast cancer	Enlarged nodules with blood and serous discharge	NA	CT/MR: multiple subcutaneous soft tissue masses	NA	Doxorubicin, ifosfamide, and radiotherapy
AlSubait et al. (2021) [53]	Case report	Sigmoid colon adenocarcinoma	Solitary, firm, asymptomatic pink nodule	Polymorphic vessels (dotted, linear, and serpentine) and a white structureless area on a pink-to-red background	NA	Tumor cells with clear cytoplasm occupying the dermis. IHC: CDX2+, CK7−, CK20+	Incisional biopsy. Patient refusal to exeresis
Avecillas-Chasin et al. (2015) [10]	Retrospective review	Meningioma	Round, firm lesions	NA	CT/MR: extra-axial enhancing mass; “hourglass” configuration with subgaleal extension	Meningioma with loss of architecture infiltrating the soft tissues. IHC: Ki67 10%	Surgical excision. Radiotherapy. Temozolamide
Çetinarslan et al. (2020) [54]	Case report	Sarcomatoid renal cell carcinoma	Violaceous-erythematous, scaly, subcutaneous nodule, with telangiectasias	White scale, polymorphic vessels (linear and hairpin), and yellowish structureless area on a light red background	NA	Tumor cells fill the dermis, with clear cytoplasm, vesicular nuclei, nucleoli-specific cells, and a tendency to spindle. IHC: CD10+, CK PAN-AE1-AE3+, PAX8+, vimentin+	Pazopanib + everolimus
Dai et al. (2023) [24]	Case report	Cervical cancer	White, discrete with a rubbery consistency nodule, fixed to the skin	NA	NA	IHC: p16+	Cadonilimab and palliative chemotherapy
Dika et al. (2020) [25]	Review	Breast cancer	Erythematous, well-defined plaques	Irregular vascular pattern, hair loss, and “macrocomedo-like” black dots	NA	NA	Surgical excision
Doan et al. (2022) [76]	Retrospective review	Colorectal adenocarcinoma, upper gastrointestinal tract tumors, pancreatic tumors, lung carcinomas, melanoma, diffuse large B-cell, lymphoma, squamous cell carcinoma from the skin of the ear canal, alveolar soft part sarcoma	NA	NA	NA	*Melanoma*: amelanotic tumor cells with eccentric nuclei and prominent nucleoli. IHC: pan-melanoma cocktail+, SOX10+, pan-CK−	NA
Huang et al. (2023) [26]	Case report	Follicular thyroid carcinoma	Large growing mass	NA	PET/CT and MR: irregular solid-cystic mass displaying extensive bony destruction	Sheets of cohesive, small, round, uniform cells intermingled with abundant capillaries and vessels; follicular and microfollicular patterns; subtle pink-red colloid-like secretions in the lumen. IHC: Vimentin+, TTF1+, Napsin A−, PAX8+, thyroglobulin+, Ki-67 10–20%	Surgical excision
Lee et al. (2023) [73]	Case report	Pulmonary large cell neuroendocrine carcinoma	Round and firm mass, with a hemispherical, raised protrusion, light pink in color, without any associated pain or tenderness, and exhibited ulceration	NA	NA	Intradermal-cord-like tumor invasion. IHC: CK7+, EMA+, CEA+, S100−, melanA−, CK20, ER−, PR−, TTF1−, napsin A−, CK5/6−	Cisplatin, gemcitabine
Lim et al. (2021) [60]	Review	Breast cancer, lung cancer, gastrointestinal tract cancer, melanoma	Destroyed hair follicles, indurated skin, and telangiectasia	NA	PET/CT: FDG-avid skin thickenings or subcutaneous nodules	NA	NA
Oh et al. (2023) [72]	Case report	Prostate cancer	Multiple red, non-tender, dome-shaped papules, and nodules	NA	NA	Sheet-like infiltration of small crowded acini with round monomorphic nuclei in the reticular dermis. IHC: PSA+, P504S+	Leuprolide acetate, abiraterone acetate
Paolino et al. (2019) [17]	Review article	Breast, thyroid, gastrointestinal, kidney, lung, uterus, central nervous system tumors	Localized asymptomatic red-violaceous nodules	NA	NA	NA	NA
Pipal et al. (2023) [20]	Case report	Ovarian cancers	Soft to firm, non-tender, fixed nodular swelling	NA	NA	Large, singly scattered, and diffusely infiltrating tumor cells	Chemotherapy
Quijano Moreno et al. (2022) [27]	Short article	Mesothelioma	Subcutaneous lesion, non-ulcerated, mobile, and painful	NA	NA	Tubules and strands through the proliferation of loosely cohesive atypical epithelioid cells with eosinophilic cytoplasm, prominent nucleoli and mild nuclear pleomorphism, with the presence of mitotic figures and apoptotic bodies. IHC: CK5/6+, WT1+, S100−, MelanA−, HMB45−, CK7−, CD31−, CD34−	Surgical excision
Ravaioli et al. (2019) [55]	Case report	Brest cancer	Multiple, well-demarcated, nonulcerated, erythematous, alopecic plaques	Erythema, erosions, peripheral black dots, “macrocomedo-like” structures, atypical vascular pattern, with dilated, serpentine and polymorphic vessels	NA	NA	NA
Riahi et al. (2012) [2]	Review	Bladder, breast, lung, rectal, renal cancers	Nodules, cystic lesions, or erythematous plaques. Asymptomatic or painful. Flesh-colored or red	NA	NA	NA	NA
Richmond et al. (2010) [37]	Review	Melanoma, breast, ovarian, lung, colon, oral, renal cell, and gastric cancers	*Melanoma*: firm dermal or subcutaneous nodules, skin-colored or faintly erythematous, visible pigment. *Others:* Skin-colored, erythematous, or purple dermal or subcutaneous nodules, or erythematous patches or plaques. Sometimes hemorrhagic, ulcerated, or with a zosteriform pattern.	NA	NA	NA	NA
Rudnicka et al. (2023) [15]	Review	Lung, prostate, breast cancers	NA	Polymorphic vessels, white structureless area, pink background	NA	NA	NA
Salari et al. (2019) [28]	Case report	Pancreatic adenosquamous carcinoma	Violaceous nodule	NA	NA	Solid nests of tumor cells with abundant eosinophilic cytoplasms and poorly formed ductal structures were identified. IHC: CK5/6+, p63+, EMA+, CK19+, CA 19−9+, CK7+, CEA+.	FOLFIRINOX, gemcitabine, paclitaxel
Sallman et al. (2020) [29]	Case report	Cholangiocarcinoma	Indurated pink nodular plaque with hemorrhagic crust and smaller satellite pink papule	NA	NA	Dermis was replaced by an infiltrative poorly differentiated carcinoma with an unremarkable epidermis. IHC: CK7+, CK20−	NA
Scalia et al. (2023) [30]	Case report	Anaplastic ependymoma	Subcutaneous lesion	NA	CT-PET 11C-methionine: increased uptake at the level of the lesion. MR: homogeneous contrast enhancement in T1W 3D- TFE sequences	Diffuse dermal and subdermal infiltration by poorly differentiated neoplastic cells, characterized by lobular growth patterns and focal areas of necrosis	Surgical excision + cisplatin, etoposide, cyclophosphamide
Sciscent et al. (2024) [23]	Review	Thyroid carcinoma	Slow-growing nodules, erythematous papules, and ulcerated lesions	NA	NA	NA	Surgical excision + radioactive iodine or radiation
Shastri et al. (2023) [31]	Case report	Pulmonary blastoma	Firm, immobile, and painless increasing swelling	NA	NA	Round to oval cells, with moderate nuclear pleomorphism, coarse chromatin, and tiny visible chromocenters/nucleoli, high nucleocytoplasmic ratio. IHC: β-catenin+, Ki-67 90%.	NA
Stanganelli et al. (2012) [36]	Retrospective analysis	Melanoma	NA	Structureless blue-white pigmentation and atypical vessels	NA	NA	NA
Subasinghe et al. (2015) [66]	Case report	Hepatocellular carcinoma	Non-tender, hemispherical, subcutaneous lump over the occipital region	NA	CT: destruction of the adjacent skull vault and intracranial extension but no penetration of the meninges	IHC: alpha fetoproteins+, Hep par1+	Surgical excision
Tung-Hahn et al. (2024) [21]	Case report	Small bowel NET	Flesh-colored subcutaneous papule	NA	NA	Well-circumscribed and focally infiltrative dermal nodule composed of closely packed nests and ductal appearing/pseudo rosetting structures. Tumor cells were cuboidal to columnar with ample amounts of amphophilic cytoplasm, round nuclei, and powdery chromatin. IHC: INSM1+, CDX2+	Surgical excision
Vezzoni et al. (2021) [16]	Review	Breast cancer	Single or multiple reddish painless patches/plaques, or flesh-colored nodules	Giant un-focused arborizing vessels and fine telangiectasias on a pink-whitish background and a single well-defined orangish lesion with polymorphic vessels surrounded by a yellow-white crust	NA	NA	Radiotherapy, chemotherapy, and hormonal therapy
Wang et al. (2014) [22]	Case report	Colonic NET	Multiple reddish papules/nodules	NA	MR: multiple space-occupying lesions with a rich blood supply in the soft tissue	Irregular and small to medium-sized tumor cells with scanty cytoplasm, hyperchromatic nuclei and distinct nucleoli in some cells, arranged in diffuse and nesting patterns in the subcutis. IHC: Syn+, CDX2+, CD56+	Etoposide, cisplatin
Wu et al. (2019) [64]	Case report	Anaplastic oligodendroglioma	Subcutaneous mass	NA	MR: homogeneously marked enhancing nodular lesion with restricted diffusion in the subcutaneous tissue	Sheets of tumor cells with round nuclei and perinuclear haloes. Necrotic areas showed increased cellularity, cellular pleomorphism, and necrotic foci. IHC: glial fibrillary acidic protein+, Olig-2+, Syn+, EGFR+, ATRX+	Surgical excision
Wu et al. (2023) [35]	Case report	Adrenocortical carcinoma	Soft, sharply demarcated, round subcutaneous nodule	NA	CT: subcutaneous soft tissue mass	IHC to exclude non-adrenocortical tumors with similar histological features	Surgical excision
Yan et al. (2022) [47]	Case report	Hepatocellular carcinoma	Exophytic nodule	NA	NA	Highly atypical cells	NA
Yuen et al. (1998) [32]	Case report	Placental site trophoblastic tumor	Non-cicatricial, alopecic patches with slight erythema and elevated plaques	NA	NA	Diffuse dermal infiltrate of cords and sheets of large, pleomorphic, polyhedral cells with abundant eosinophilic cytoplasm. Destruction of hair follicles and extensive deposition of fibrinoid material. IHC: human placental lactogen+, CK+	Etoposide, methotrexate, actinomycin D, cyclophosphamide, vincristine
Zhang et al. (2023) [33]	Case report	Lung carcinoma	Motionless, skin-colored, without hair, painless mass	NA	MR: soft tissue nodule	IHC: panCK+, CK7+, CD56+, P40+, NapsinA−	Paclitaxel and xindirizumab

CT: computed tomography; ER: estrogen receptor; FDG: fluorodeoxyglucose; IHC: immunohistochemistry; MR: magnetic resonance; NA: not acquired; PET: positron emission tomography; PR: progesterone receptor.
